# Field Efficacy of *Metarhizium robertsii* (LCM S15) for Controlling Free-Living Stages of Gastrointestinal Nematodes in Goats

**DOI:** 10.3390/pathogens15060594

**Published:** 2026-06-01

**Authors:** Ially de Almeida Moura, Antônio Wesley Oliveira da Silva, Gabriel da Silva Correia, Giancarlo Bomfim Ribeiro, Mayara Macêdo Barrozo, Thaís Almeida Corrêa, Patrícia Silva Gôlo, Isabele da Costa Ângelo, Caio Márcio de Oliveira Monteiro, Éverton Kort Kamp Fernandes, Vânia Rita Elias Pinheiro Bittencourt, Alexandre Dias Munhoz, Wendell Marcelo de Souza Perinotto

**Affiliations:** 1 Programa de Pós-Graduação em Ciência Animal, Universidade Estadual de Santa Cruz, Ilhéus 45662-900, Bahia, Brazil; 2 Programa de Ciência Animal nos Trópicos, Universidade Federal da Bahia, Salvador 40170-115, Bahia, Brazil; 3 Departamento de Biociências e Tecnologia, Instituto de Patologia Tropical e Saúde Pública, Universidade Federal de Goiás, Goiânia 74605-050, Goiás, Brazil; 4 Departamento de Parasitologia Animal, Instituto de Veterinária, Universidade Federal Rural do Rio de Janeiro, Seropédica 23897-000, Rio de Janeiro, Brazil; thaisalmeida_tac@yahoo.com.br (T.A.C.); patriciagolo@gmail.com (P.S.G.);; 5 Departamento de Epidemiologia e Saúde Pública, Instituto de Veterinária, Universidade Federal Rural do Rio de Janeiro, Seropédica 23890-000, Rio de Janeiro, Brazil; isabeleangelo@yahoo.com.br

**Keywords:** biocontrol, entomopathogen, strongylids, goat farming

## Abstract

The rise in anthelmintic resistance in small ruminants has driven the search for sustainable control alternatives. Among these, entomopathogenic fungi such as *Metarhizium robertsii* stand out for their potential to reduce the free-living stages of gastrointestinal nematodes (GINs). This study evaluated the field efficacy of *M. robertsii* (LCM S15) under climatic conditions in the Recôncavo region of Bahia, Brazil. The experiment was conducted between November 2022 and July 2023 in a completely randomized design with four groups (*n* = 8): aqueous control, oil control, aqueous suspension, and oil formulation of *M. robertsii*. Egg counts per gram of feces (EPG) and L3 larval recovery were assessed by coproculture and the Baermann technique. Efficacy ranged from 15.23% to 27.34%, with the oil formulation showing higher performance. *Haemonchus* sp. and *Trichostrongylus* sp. were the most prevalent genera. These findings suggest the potential of *M. robertsii* (LCM S15) as a biological control agent under field conditions.

## 1. Introduction

Gastrointestinal nematodes (GINs) remain one of the main constraints in global goat farming, impairing zootechnical performance and animal welfare while generating significant treatment costs and production losses. Recent reports highlight the increasing occurrence of anthelmintic resistance (AR) to multiple drug classes across different continents, reinforcing the need for sustainable and integrated control strategies [1].

The Northeast region of Brazil accounts for approximately 94% of the national goat herd [2]. Within this region, the Recôncavo of Bahia, part of the Salvador Metropolitan mesoregion, is characterized by a hot and humid tropical climate [3]. Although these conditions favor ruminant production, they also provide an ideal environment for the development and persistence of parasitic stages in soil and pasture.

GIN infections represent a major health challenge, leading to weight loss, reduced milk production, and even death in untreated animals [4]. The most prevalent genera include *Haemonchus* spp., *Trichostrongylus* spp., *Cooperia* spp., *Oesophagostomum* spp., and *Strongyloides papillosus* [5,6]. Infections with more than 500 *Haemonchus* spp. may result in acute mortality, particularly after periods of drought followed by heavy rainfall [7]. Additionally, infective larvae can remain viable in the environment for up to 150 days, serving as a continuous source of reinfection [8].

In addition to the selective pressure exerted by drug use, climatic factors play a central role in the epidemiology of GINs. L3 larvae can survive for long periods in fecal pellets, ensuring reinfection even under adverse conditions [9]. Rainfall and temperature are critical for larval development and migration [10], with optimal development occurring at approximately 100% relative humidity and temperatures between 22 and 26 °C [11]. Under these conditions, larvae can persist in pasture for about 10 weeks [12], in soil for three to nine weeks [13], and in feces for 100 to 150 days [8]. They may also survive at low temperatures in pasture for up to one year [11]. Under unfavorable conditions, larvae may undergo hypobiosis within the host [11].

To control infections caused by GINs, the use of chemical anthelmintics remains the standard approach. However, inappropriate practices such as underdosing, prophylactic mass treatments, and the continuous use of the same drug classes have accelerated the development of resistance [14,15]. This leads to economic losses, as treatments become less effective and fail to improve animal health [16]. Resistance is associated with the selection of resistant parasite populations [6]. The main classes of anthelmintics include benzimidazoles (albendazole, fenbendazole, oxfendazole), avermectins (ivermectin), imidazothiazoles (levamisole), and salicylanilides (closantel) [17], with widespread resistance reported in recent studies [18,19,20,21,22]. In addition, concerns about drug residues in animal products and the environment have encouraged the search for alternative control strategies [23,24]. In this context, biological control emerges as a complementary approach aimed at improving productivity, preserving animal welfare, and reducing environmental impacts.

The use of nematophagous fungi represents a promising alternative for parasite control [25]. Recent studies using *Duddingtonia flagrans* have demonstrated significant efficacy [20,26,27,28,29,30]. Other fungi, such as *Clonostachys rosea*, *Arthrobotrys musiformis*, and *Trichoderma esau*, have also shown effectiveness in preying on *H. contortus* larvae [31].

*Metarhizium* spp. sensu lato is considered a promising candidate due to its ability to produce hydrolytic enzymes that affect a wide range of parasites [32,33,34,35,36]. In ruminants, *Metarhizium* spp. has demonstrated efficacy against arthropods in cattle [37,38], as well as potential activity against free-living stages of nematodes in horses [39] and goats [40].

Therefore, the present study aimed to evaluate, under field conditions in goats raised in the Recôncavo region of Bahia, the potential activity of *M. robertsii* (LCM S15) against free-living stages of gastrointestinal nematodes.

## 2. Materials and Methods

This study complied with the ethical principles established by Brazilian legislation (Law No. 11,794/2008; Decree No. 6899/2009) and the regulations of the National Council for the Control of Animal Experimentation (CONCEA). The experimental protocol was approved by the Animal Use Ethics Committee (CEUA) of the Federal University of Recôncavo da Bahia (UFRB) under protocol No. 23007.00008860/2021–47.

The study was conducted at the experimental farm of UFRB, located in Cruz das Almas, Bahia, Brazil, with laboratory analyses performed at the Laboratory of Parasitology and Parasitic Diseases of the University Veterinary Hospital. The site is situated at 12°40′39″ S and 39°06′26″ W, at an altitude of 226 m, and is characterized by a hot and humid tropical climate according to the Köppen classification. Meteorological data were obtained from the database of the Embrapa Cassava and Fruits Meteorological Station.

The field studies were conducted from November 2022 to March 2023 and from April to July 2023. The experimental design was completely randomized, comprising four groups of eight animals each: CTR-A (aqueous control: distilled water + 0.1% Tween 80), CTR-O (oil control: mineral oil + 0.1% Tween 80), FUN-A (aqueous suspension of *Metarhizium robertsii* LCM S15), and FUN-O (oil formulation of *M. robertsii* LCM S15). Each treatment was allocated to a single paddock under field conditions; therefore, paddock replication was not performed. Consequently, environmental characteristics specific to each paddock may have contributed to variability among treatments. Thus, the present study should be interpreted as a preliminary field trial conducted under semi-natural grazing conditions.

Each fungal formulation was prepared at a final concentration of 1 × 10^8^ conidia/mL, quantified using a Neubauer chamber under an optical microscope. Conidial viability was previously assessed, and only suspensions showing ≥95% germination after 24 h of incubation on BDA medium were considered suitable.

Treatment efficacy was calculated based on the percentage reduction of larvae, using the following formula [41]:

Percentage reduction (%) = [(mean number of larvae in control group − mean number of larvae in treated group)/mean number of larvae in control group] × 100

Negative efficacy values observed when treated groups presented higher mean counts than the respective control groups were expressed as 0%, since negative reductions were considered biologically non-interpretable for the purposes of efficacy presentation under field conditions.

The study included 32 female crossbred goats (*Capra hircus*), aged 6 to 24 months, with an average body weight of 30 kg, from the UFRB experimental herd. Fifteen days prior to the start of the experiment, all animals were treated with albendazole; however, complete elimination of fecal egg shedding was not achieved. Therefore, residual EPG values were still observed in some animals before the beginning of the experimental period.

The animals were allocated to experimental groups according to their respective treatments, considering the post-treatment EPG values in order to obtain a homogeneous distribution of infection levels among groups before the beginning of fungal applications, from November 2022 to March 2023 and from April to July 2023. Throughout the experimental period, they received a concentrate diet composed of 75% finely ground corn and 25% soybean meal, supplied daily at 1% of body weight, in addition to supplementation with 5 g/day of Organew^®^ (Organnact, Curitiba, Brazil). Water and mineral salt were provided ad libitum.

Animal health was monitored weekly through general clinical examinations and laboratory analyses. In cases of pale mucous membranes associated with high egg counts per gram of feces (EPG), animals were treated according to institutional health protocols.

For this study, *Metarhizium robertsii* (LCM S15) was used. Conidia were produced on a rice-based substrate following a method previously described in the literature [32], using 40 kg of type 1 polished white rice (Camil^®^, Camil Alimentos S.A.—Rio Grande do Sul, Brazil). Initially, the rice was placed in a container and soaked in water until fully submerged for 20 min at room temperature. The excess water was then drained, and the moist rice was transferred to polypropylene plastic bags, with approximately 500 g per bag.

The bags containing the rice were autoclaved at 120 °C for 20 min. After sterilization, the bags were placed on a bench previously disinfected with 70% alcohol and allowed to cool at room temperature for approximately one hour. A conidial suspension (~1.5 × 10^8^ conidia/mL) was then prepared, and 20 mL of this suspension was inoculated into each bag using a sterile syringe.

The bags were manually shaken to ensure homogeneous distribution of the conidia throughout the rice substrate and subsequently incubated in a B.O.D. (Biochemical Oxygen Demand) chamber at 25 ± 1 °C and relative humidity ≥ 80% for seven days.

The following treatments were applied. An aqueous solution without fungal conidia, consisting of sterile distilled water and 0.1% Tween 80, was prepared and used for both the aqueous control group and as the base for the aqueous treatment. For the oil control group, a formulation containing sterile distilled water, 0.1% Tween 80, and 10% mineral oil (Fertiliza^®^, Avaré, São Paulo, Brazil) was prepared.

The formulations used for the *Metarhizium robertsii* (LCM S15) aqueous and oil treatments were obtained by washing the colonized rice substrate. Briefly, the rice was placed in buckets containing a solution of sterile distilled water and 0.1% Tween 80, homogenized, and subsequently filtered through a sieve to recover the conidial suspension. For the oil formulation, 10% mineral oil was added to the conidial suspension. All formulations were quantified under an optical microscope using a Neubauer chamber and adjusted to a concentration of 1 × 10^8^ conidia/mL. Conidial viability was assessed based on germination rates, as previously described [32].

Each group of animals was maintained in a separate paddock. The paddocks assigned to the aqueous control and aqueous fungal suspension groups measured 0.83 ha, while those assigned to the oil control and oil fungal suspension groups measured 0.88 ha and 0.71 ha, respectively. All paddocks were composed of *Urochloa (Brachiaria) decumbens* pasture and had been previously grazed by naturally infected animals for a period of 60 days to ensure pasture contamination before the introduction of the experimental animals.

Treatment applications were performed at a rate of 90 L/ha using a manual backpack sprayer (Vonder^®^, Curitiba, Paraná, Brazil) [32]. Applications were carried out at three times: day 0 (the day animals were introduced into the paddocks) and two additional applications at 21-day intervals.

Soil samples were collected from each paddock, considering four quadrants, with each sample composed of five randomly selected points within each quadrant. Sampling was performed at the following time points: on day 0 (before treatment application) to verify the natural presence of *Metarhizium* sp. in the soil; on day 20 (before the second application); on day 41 (before the third application); and on days 63 and 102 after the initial treatment.

Soil samples were stored under refrigeration and subsequently processed. For each composite sample, a 0.35 g aliquot was weighed into microtubes and diluted in 1 mL of 0.01% Tween 80 solution. After vigorous agitation for 30 s using a vortex mixer, a 50 µL aliquot was plated onto Petri dishes (three plates per sample) containing CTC selective medium (yeast extract, chloramphenicol, thiabendazole, and cycloheximide). The suspension was evenly spread over the culture medium using a Drigalski spatula (São Paulo, Brazil), following a previously described methodology [42].

The plates were incubated in a B.O.D. chamber at 25 ± 1 °C for 21 days and evaluated every seven days for the presence of colonies characteristic of *Metarhizium* sp. Macro- and microscopic characteristics were assessed according to the literature [43,44,45]. Colonies were expected to show an initial white pigmentation, turning yellow and then green as they matured. Microscopically, conidia were expected to be cylindrical to oval, slightly constricted in the middle, sometimes truncated at both ends, measuring approximately 3.5–9.0 µm in length and arranged in columnar chains. Identification was based exclusively on macro- and microscopic morphological characteristics compatible with *Metarhizium* spp., and no molecular analyses were performed to confirm isolate identity.

Fecal samples were collected from all animals in each group every seven days, directly from the rectal ampulla. Egg counts per gram of feces (EPG) were determined according to a previously described method [46]. Every 21 days, coprocultures were prepared using 20 g of feces mixed with sawdust and maintained at room temperature for 10 days to obtain infective larvae, which were subsequently identified according to the literature [47].

**Figure 1:**
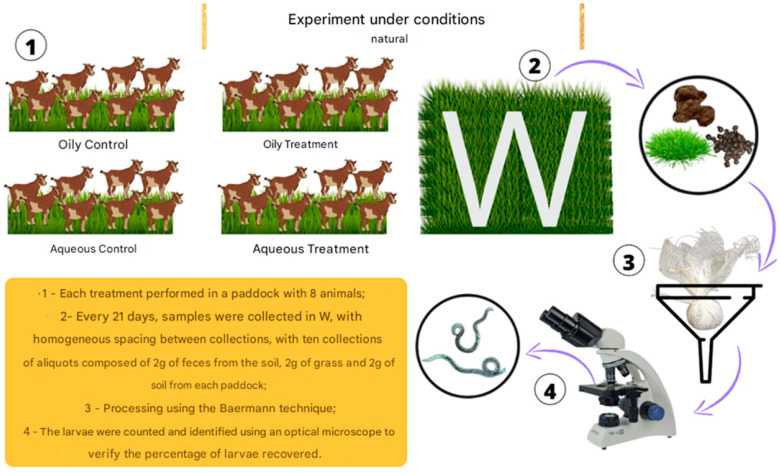
Schematic representation of sample collection for the Baermann technique.

Additionally, every 21 days, 2 g aliquots of fecal samples were collected from the paddocks using a W-shaped sampling pattern, with uniform spacing between collection points, a strategy adapted from methodologies previously used for quantification of infective gastrointestinal nematode larvae in pasture [48]. Each sample was considered one replicate and processed using the Baermann technique. Briefly, samples were placed in gauze folded into four layers and positioned in a funnel connected to a rubber tube at its lower end. The tube was clamped, and warm water (45 °C) was added until it contacted the fecal material. After standing overnight, the clamp was released, and approximately 7 mL of sediment was collected into centrifuge tubes, with the volume adjusted to 12 mL with water. Samples were centrifuged at 1120× *g* for two minutes. The supernatant was discarded, leaving approximately 2 mL, which was homogenized prior to larval identification and counting under an optical microscope to determine the percentage of larvae recovered (Figure 1).

Statistical analysis was performed considering the longitudinal monitoring of parasitological parameters under field conditions. Data were initially evaluated for normality using the Shapiro–Wilk test. When normality assumptions were met, analysis of variance (ANOVA) followed by Tukey’s test was applied. Non-parametric data were analyzed using the Kruskal–Wallis test followed by pairwise comparisons.

The individual animal was considered the observational unit for parasitological evaluations, and repeated measurements were obtained from the same animals throughout the experimental period. Due to the exploratory field design and absence of paddock replication, the statistical analyses and interpretation of treatment effects were performed with caution.

All analyses were conducted at a significance level of 5% (*p* ≤ 0.05).

## 3. Results

### 3.1. Viability of Conidial Suspensions

Conidia from *Metarhizium* spp. suspensions used in both in vitro and field experiments showed a germination rate of 99% at an incubation temperature of 25 ± 1 °C.

### 3.2. Field Experimental Assay—First Period

No significant differences were observed among the mean EPG values of the experimental groups during the first six sampling points. However, from the seventh sampling point—corresponding to 21 days after the second application, statistically significant differences were detected (*p* < 0.05). At this point, the oil formulation of *Metarhizium robertsii* (LCM S15) maintained lower mean EPG values compared to the oil control group, while the aqueous suspension of *M. robertsii* (LCM S15) showed lower mean values compared to the aqueous control group (Table 1).

**Figure 2:**
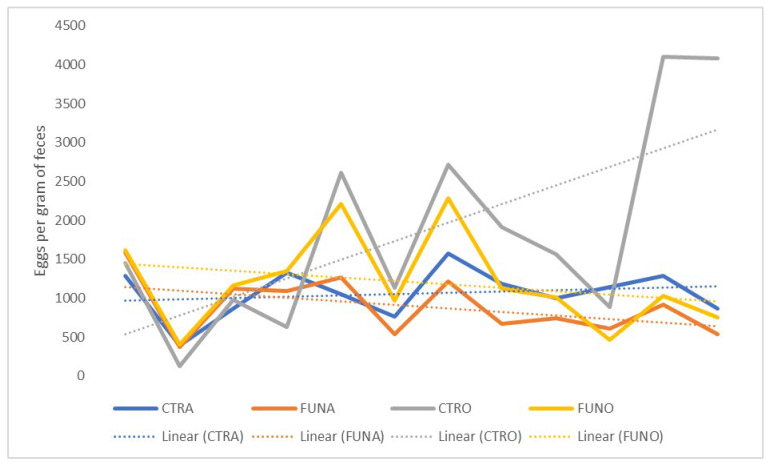
Mean egg counts per gram of feces (EPG) in goats subjected to different treatments over 12 sampling points: aqueous control (CTR-A), oil control (CTR-O), aqueous suspension of *Metarhizium robertsii* (LCM S15) (FUN-A), and oil formulation of *M. robertsii* (LCM S15) (FUN-O). Dashed lines represent the linear trends of the respective groups.

In Figure 2, it can be observed that over the twelve sampling points, the control groups showed an increasing trend in egg shedding, particularly the oil control group, which exhibited a marked rise from the fifth sampling onward, reaching the highest values in the final collections. In contrast, treatments with *Metarhizium robertsii* (LCM S15) showed lower values throughout most of the experimental period, with a decreasing linear trend. These results indicate that the treatments were effective in reducing the shedding of gastrointestinal nematode eggs compared to the control groups.

The aqueous suspension of *Metarhizium robertsii* (LCM S15) showed efficacy ranging from 0 to 46.1%, whereas the oil formulation ranged from 0 to 81.6% compared to their respective control groups. The mean efficacy of the aqueous suspension was 19.84%, while the oil formulation reached 27.24% when compared to the aqueous and oil control groups, respectively (Table 2).

When the number of gastrointestinal nematode (GIN) larvae was evaluated using the Baermann technique, the following results were observed: groups treated with the aqueous suspension and oil formulation of *M. robertsii* (LCM S15) showed statistically significant differences compared to their respective control groups, except at the second sampling point, where the aqueous suspension of *M. robertsii* (LCM S15) did not differ from the aqueous control group. In contrast, the group treated with the oil formulation of *M. robertsii* (LCM S15) differed significantly from all groups across the three sampling points (Table 3). The efficacy of the aqueous suspension and oil formulation of *M. robertsii* (LCM S15) was 15.75% and 27.34%, respectively (Table 4).

**Figure 3:**
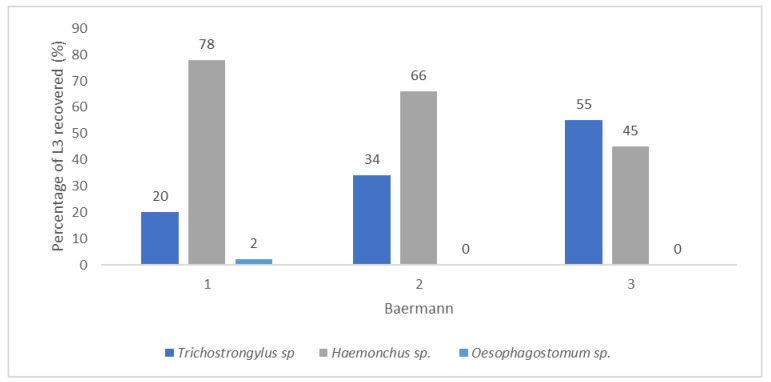
Percentage of infective larvae (L3) of gastrointestinal nematodes recovered using the Baermann technique, corresponding to *Trichostrongylus* sp., *Haemonchus* sp., and *Oesophagostomum* sp., across the three sampling points evaluated.

**Figure 4:**
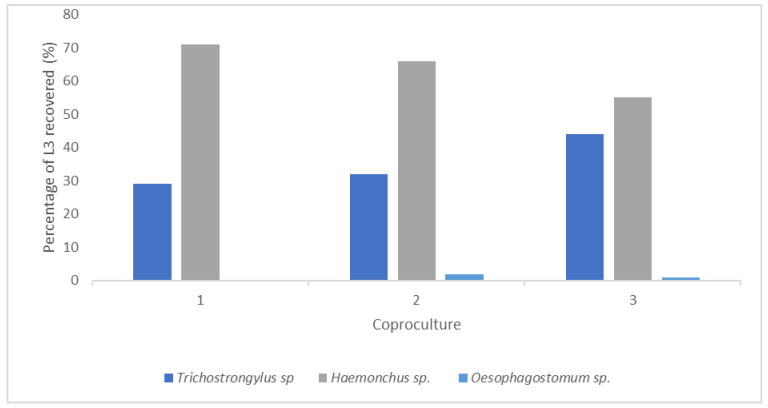
Percentage of infective larvae (L3) of gastrointestinal nematodes recovered using the coproculture technique, corresponding to *Trichostrongylus* sp., *Haemonchus* sp., and *Oesophagostomum* sp., across the three sampling points evaluated.

Across the three evaluations performed using the Baermann technique, *Haemonchus* sp. and *Trichostrongylus* sp. were the most prevalent genera. *Haemonchus* sp. predominated in the first two sampling points, whereas *Trichostrongylus* sp. was the most prevalent in the third. Additionally, a prevalence of 2% for *Oesophagostomum* sp. was recorded in the first sampling point (Figure 3). In the coprocultures, *Haemonchus* sp. was the most prevalent genus in all three sampling points (Figure 4).

Meteorological data showed daily variations throughout the study period. Accumulated precipitation ranged from 62.6 mm to 185.7 mm, with November presenting the highest rainfall, despite typically corresponding to the dry season in the region. During this month, intense rainfall events were recorded on only nine sampling days.

Relative humidity reached maximum values ranging from 82.6% to 98.7%. The lowest mean values were observed in November and December, whereas the highest occurred between January and March. Minimum values ranged from 45.4% to 59.2%, with February showing the lowest mean and November the highest.

Mean minimum and maximum temperatures remained stable throughout the experimental period, ranging from 22.0 °C to 22.8 °C and from 28.1 °C to 31.3 °C, respectively.

### 3.3. Field Experimental Assay—Second Period

In the evaluation of egg counts per gram of feces (EPG), no significant differences were observed among the groups during the first six sampling points. From the seventh sampling point onward, the group treated with the oil fungal formulation (FUN-O) showed lower values compared to the aqueous control group (CTR-A), while the aqueous fungal suspension (FUN-A) and oil control (CTR-O) exhibited intermediate values, not differing statistically from the other groups. At the tenth sampling point, the CTR-A group presented the highest mean EPG, differing significantly from FUN-A, CTR-O, and FUN-O, which showed lower and statistically similar values among themselves (Table 5).

**Figure 5:**
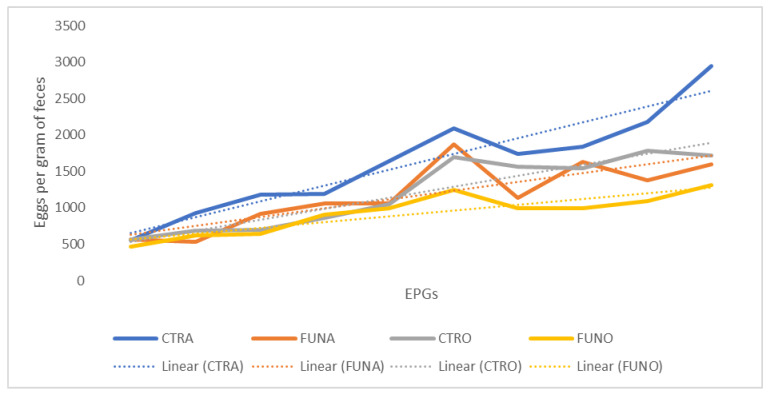
Evolution of egg counts per gram of feces (EPG) in goats treated with aqueous fungal suspension (FUN-A), oil fungal formulation (FUN-O), and their respective controls (CTR-A and CTR-O), across ten consecutive sampling points. Solid lines represent the observed mean values for each group, while dashed lines indicate the linear trend of the data.

In Figure 5, it can be observed that all four groups showed an increasing trend in mean values over the course of the experimental period. However, despite this overall increase, the groups treated with the aqueous suspension and oil formulation of *Metarhizium robertsii* (LCM S15) maintained lower mean values compared to their respective control groups.

The aqueous suspension of *M. robertsii* (LCM S15) showed efficacy ranging from 0% to 45.76%, whereas the oil formulation ranged from 0% to 39.16% compared to the aqueous and oil control groups, respectively. The mean efficacy of the aqueous suspension and oil formulation of *M. robertsii* (LCM S15) was 24.79% and 16.54%, respectively, when compared to their respective control groups (Table 6).

In the Baermann assays, no significant differences were observed among the groups in the first two evaluations. Only in the third evaluation did the group treated with the oil formulation of *M. robertsii* (LCM S15) show a significant difference compared to the aqueous control group, with no differences observed among the other groups (Table 7). The mean efficacy of the aqueous and oil formulations of *M. robertsii* (LCM S15) was 15.23% and 21.12%, respectively (Table 8).

**Figure 6:**
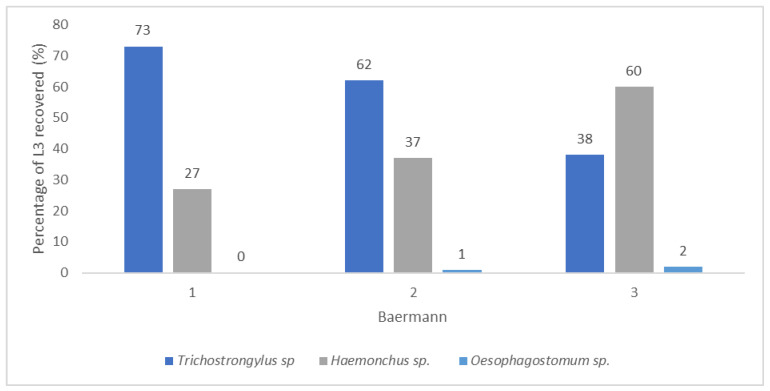
Percentage of infective larvae (L3) of gastrointestinal nematodes recovered using the Baermann technique, corresponding to *Trichostrongylus* sp., *Haemonchus* sp., and *Oesophagostomum* sp., across the three sampling points evaluated.

Across the three Baermann evaluations, *Haemonchus* sp. and *Trichostrongylus* sp. were the most prevalent genera. *Trichostrongylus* sp. showed the highest prevalence in the first two evaluations, with 73% and 62%, respectively, whereas *Haemonchus* sp. was the most prevalent in the final evaluation, reaching 60%. Additionally, a prevalence of 1% for *Oesophagostomum* sp. was recorded in the last sampling point (Figure 6).

**Figure 7:**
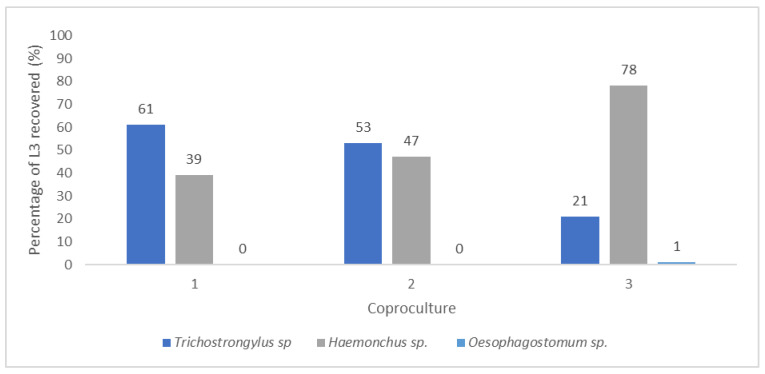
Percentage of infective larvae (L3) of gastrointestinal nematodes recovered using the coproculture technique, corresponding to *Trichostrongylus* sp., *Haemonchus* sp., and *Oesophagostomum* sp., across the three sampling points evaluated.

In the coprocultures, *Haemonchus* sp. was the most prevalent genus only in the final sampling point, with a prevalence of 78%, whereas *Trichostrongylus* sp. predominated in the first two sampling points, with prevalences of 61% and 53%, respectively. Additionally, *Oesophagostomum* sp. showed prevalences of 1% and 2% in the last two sampling points, respectively (Figure 7).

Meteorological data showed daily variations throughout the experimental period. Rainfall exhibited monthly fluctuations, ranging from 26.2 mm to 136.6 mm, with June presenting the highest precipitation during the study. Relative humidity remained stable in terms of maximum mean values, ranging from 83.9% to 90.0%, with the highest value also recorded in June. Minimum humidity ranged from 55.8% to 76.5%, with April showing the lowest means and May the highest. Regarding temperature, no significant variations were observed in monthly means, with minimum temperatures ranging from 19.2 °C to 22.3 °C and maximum temperatures from 24.9 °C to 29.0 °C.

### 3.4. Detection of Colonies Morphologically Compatible with Metarhizium sp. in Soil

Colonies morphologically compatible with Metarhizium sp. were isolated from soil samples collected in the experimental paddocks at different evaluation periods. The isolated colonies exhibited macroscopic morphological characteristics typical of the genus *Metarhizium*, initially white, later turning yellow, and eventually developing a green coloration upon maturation. Microscopic characteristics were also consistent with the genus, presenting cylindrical to oval conidia, slightly constricted at the middle, and possibly truncated at both ends, arranged in column-like chains, as previously described by other authors [43,44,45]. No colonies with morphological characteristics of the genus *Metarhizium* were observed in samples collected on day 0, suggesting the absence of colonies morphologically compatible with Metarhizium sp. prior to treatment application. Likewise, no colonies with characteristics of *Metarhizium* were detected in the control groups throughout the experimental period. However, since molecular analyses were not performed, it was not possible to confirm whether the recovered colonies corresponded specifically to *M. robertsii* (LCM S15).

## 4. Discussion

The present study suggests, for the first time under field conditions, the ability of *Metarhizium robertsii* (LCM S15) to reduce the free-living stages of gastrointestinal nematodes (GINs) in goats, confirming the potential of this fungus as a biological control agent in animal production systems. The experimental design adopted in the present study was adapted from previous field studies evaluating entomopathogenic fungi against livestock parasites under natural grazing conditions. Nevertheless, the absence of paddock replication should be considered when interpreting treatment effects.

Although the specific mechanisms of penetration into helminths have not yet been fully demonstrated, it is likely that they involve the secretion of hydrolytic enzymes, such as proteases of the Pr1 family and aminopeptidases, which degrade the collagenous matrix of the tegument [48]. Thus, the infective behavior observed in this study against goat gastrointestinal nematodes may occur through mechanisms like those reported in ticks and insects [48,49], reinforcing the potential role of this fungus as an alternative tool in integrated parasite management in goats.

Coproculture and Baermann analyses revealed the presence of three genera: *Haemonchus* sp., *Trichostrongylus* sp., and *Oesophagostomum* sp. These findings are consistent with previous studies conducted in the region [50], which also reported a higher prevalence of *Haemonchus* sp. and *Trichostrongylus* sp. [50]. Among these, *Haemonchus contortus* and *Trichostrongylus* spp. are recognized as the main contributors to production losses in goat farming [51]. *H. contortus*, being a hematophagous parasite, can cause severe anemia [4], while *T. colubriformis* causes persistent infections in the small intestine [52], leading to economic losses due to poor growth and diarrhea [11]. In this study, *M. robertsii* (LCM S15) reduced the occurrence of these genera, suggesting a potential reduction in pasture reinfection pressure and, consequently, improved zootechnical performance.

In the present study, the aqueous suspension of *M. robertsii* (LCM S15) reduced the number of infective larvae in both experimental periods, with mean efficacies of 19.84% and 24.79%, respectively. The oil formulation showed mean efficacy of 27.24% and 16.54% in the first and second periods, respectively. Although moderate, these reductions are biologically relevant in tropical environments, where parasite challenge is constant and reinfection occurs rapidly. Previous studies have demonstrated that isolates of *M. anisopliae* can reduce free-living stages of nematodes in horses [41] and goats [40]. Thus, the performance of *M. robertsii* LCM S15 is consistent with the literature on the genus *Metarhizium*, suggesting that its action on helminths may follow a similar pattern, even under field conditions.

The oil formulation of *M. robertsii* LCM S15 showed higher efficacy at several evaluation points, However, these effects cannot be attributed exclusively to fungal activity, since mineral oil itself may have influenced environmental conditions affecting larval survival and conidial persistence under field conditions, possibly due to the protection conferred to conidia against environmental stress [53,54], as well as the cutinophilic properties of the oil [55], which may enhance conidial adhesion to nematodes. This is particularly relevant considering that nematodes possess a body wall composed of hypodermis, muscle layer, and an external cuticle [39]. These results are consistent with previous studies [40] and with reports in ticks, where oil-based formulations increased fungal efficacy [37,54,56,57,58,59].

The effects observed in the oil control group and in the oil formulation of *M. robertsii* LCM S15 may also be associated with the direct action of mineral oil on larvae. The higher EPG values observed in the CTR-O group during part of the first experimental period may be associated with environmental variability among paddocks, differences in pasture contamination dynamics. Studies with ticks have shown that oil-based formulations may cause mortality by asphyxiation due to blockage of respiratory spiracles [60]. A similar mechanism may be considered for GINs, as the oil could interfere with gas exchange through the cuticle.

Climatic factors may have directly influenced fungal performance. Rainfall varied considerably between experimental periods (26–185 mm). Although November is typically a dry month in the region, it presented the highest rainfall during the study. Conversely, April and July, typically part of the rainy season, showed lower precipitation, indicating that local rainfall patterns do not follow well-defined dry and wet seasons. These conditions may have affected conidial dispersal and survival in soil. Heavy rainfall may have caused conidial leaching, temporarily reducing their density in pastures, whereas high humidity conditions may have favored germination and infection [32,61]. Temperature fluctuations may also act as stress factors for conidia [62]; however, recorded temperatures (19–31 °C) remained within the optimal range for *Metarhizium* spp. development [63], and relative humidity values (82.6–98.7%) provided suitable conditions for fungal activity, as high humidity is essential for its development [61].

In agriculture, fungal biological control is already recognized as an important tool for integrated pest management and sustainable production systems worldwide [64]. In veterinary medicine, it has been increasingly studied; however, until effective fungal-based products become widely available, control of ticks and helminths still relies mainly on synthetic chemical compounds [65]. Similarly, in GIN control, integrated approaches remain essential. Studies indicate that even under global anthelmintic resistance, combinations such as levamisole and albendazole may still represent therapeutic options [1].

The efficacy values observed in the present study were moderate and variable throughout the experimental period, suggesting potential activity of *M. robertsii* under field conditions rather than definitive field efficacy. Although colonies morphologically compatible with *Metarhizium* spp. were recovered from treated paddocks throughout the experimental period, molecular analyses were not performed to confirm the persistence of the specific isolate *M. robertsii* (LCM S15) in soil samples.

Future studies may evaluate the virulence of *M. robertsii* against pre-parasitic stages of ruminant GINs in association with different adjuvants such as vegetable oils. Additionally, different oil concentrations could be tested to optimize formulation cost and efficacy. Field evaluations using different fungal doses may also support its application in integrated parasite management systems targeting multiple parasites, since *Metarhizium* spp. is also known to be pathogenic to other ruminant parasites such as *Rhipicephalus microplus*.

## Figures and Tables

**Table 1:** Mean ± standard deviation of egg counts per gram of feces (EPG) after semi-natural conditions testing with the following treatments: aqueous control (CTR-A), oil control (CTR-O), aqueous suspension of *Metarhizium robertsii* (LCM S15) (FUN-A), and oil formulation of *M. robertsii* (LCM S15) (FUN-O), during the period from November to February.

	Treatments			
	CTR-A	FUN-A	CTR-O	FUN-O
1° EPG	1287.5 ± 644.6 a	1587.5 ± 1133.1 a	1450.0 ± 719.1 a	1612.5 ± 1200.5 a
2° EPG	387.5 ± 513.9 a	375.0 ± 599.4 a	125.0 ± 128.1 a	400.0 ± 552.9 a
3° EPG	862.5 ± 1040.5 a	1125.0 ± 1234.9 a	975.0 ± 894.0 a	1162.5 ± 879.8 a
4° EPG	1325.0 ± 1605.1 a	1087.5 ± 1885.6 a	633.3 ± 686.0 a	1350.0 ± 1108.4 a
5° EPG	1050.0 ± 891.2 a	1262.5 ± 1494.6 a	2616.6 ± 3345.6 a	2212.5 ± 1841.9 a
6° EPG	762.5 ± 671.0 a	537.5 ± 858.4 a	1133.3 ± 631.4 a	962.5 ± 879.8 a
7° EPG	1575.0 ± 2523.4 ab	1212.5 ± 2241.4 b	2716.6 ± 1894.6 a	2287.5 ± 3391.3 ab
8° EPG	1187.5 ± 984.7 ab	671.4 ± 1041.9 b	1916.6 ± 1105.2 a	1125.0 ± 931.5 ab
9° EPG	1000.0 ± 841.7 a	737.5 ± 851.7 a	1566.6 ± 1230.7 a	1012.5 ± 1619.9 a
10° EPG	1137.5 ± 686.4 a	612.5 ± 540.9 ab	883.3 ± 780.8 ab	462.5 ± 410.3 a
11° EPG	1287.5 ± 687.5 ab	912.5 ± 1078.9 b	4100.0 ± 3688.9 a	1025.0 ± 936.1 b
12° EPG	862.5 ± 534.3 b	537.5 ± 440.5 b	4083.3 ± 3407.2 a	750.0 ± 602.3 b

Means followed by different letters in the same row indicate significant differences (*p* < 0.05), according to Student’s *t*-test.

**Table 2:** Efficacy (%) of *Metarhizium robertsii* (LCM S15) in aqueous (FUN-A) and oil (FUN-O) formulations, compared to the aqueous (CTR-A) and oil (CTR-O) control groups under field conditions from November 2022 to March 2023.

	Efficacy	
	FUN-A	FUN-O
1° EPG	0%	0%
2° EPG	3.2%	0%
3° EPG	0%	0%
4° EPG	0%	0%
5° EPG	0%	15.4%
6° EPG	29.5%	15%
7° EPG	23%	15.7%
8° EPG	43.4%	41.3%
9° EPG	26.2%	35.3%
10° EPG	46.1%	47.6%
11° EPG	29.1%	75%
12° EPG	37.6%	81.6%
Mean efficacy (%)	19.84%	27.24%

**Table 3:** Mean ± standard deviation of the number of gastrointestinal nematode (GIN) larvae recovered from goats using the Baermann technique across three sampling points, under different treatments: aqueous suspension of *Metarhizium robertsii* (LCM S15) (FUN-A), oil formulation of *M. robertsii* (LCM S15) (FUN-O), aqueous control (CTR-A), and oil control (CTR-O).

		Number of Larvae	
Groups	1° Baermann	2° Baermann	3° Baermann
FUN-A	121.6 ± 42.9 a	137.8 ± 77.5 ab	59.9 ± 21.1 ab
CTR-A	91.3 ± 38.6 a	163.5 ± 103.1 ab	87.9 ± 38.8 a
FUN-O	132.1 ± 60.2 a	94.2 ± 46.2 b	36.3 ± 24 b
CTR-O	93.8 ± 24.1 a	151.9 ± 58.3 a	64.9 ± 32.8 ab

Means followed by the same letter in the column do not differ significantly (*p* ≥ 0.05, Student’s *t*-test).

**Table 4:** Efficacy (%) of *Metarhizium robertsii* (LCM S15) in aqueous (FUN-A) and oil (FUN-O) formulations against gastrointestinal nematode (GIN) larvae of goats, recovered using the Baermann technique across three consecutive sampling points, compared to control groups.

		Efficacy (%)		
Groups	1° Baermann	2° Baermann	3° Baermann	Mean Efficacy (%)
FUN-A	0%	15.41%	31.85%	15.75%
FUN-O	0%	37.98%	44.06%	27.34%

**Table 5:** Mean ± standard deviation of egg counts per gram of feces (EPG) under semi-natural conditions with the following treatments: aqueous control (CTR-A), oil control (CTR-O), aqueous suspension of *Metarhizium robertsii* (LCM S15) (FUN-A), and oil formulation of *M. robertsii* (LCM S15) (FUN-O), from April to July 2023.

	CTR-A	FUN-A	CTR-O	FUN-O
1° EPG	550.0 ± 261.8 a	562.5 ± 238.6 a	562.5 ± 373.9 a	462.5 ± 266.9 a
2° EPG	925.0 ± 716.6 a	537.5 ± 206.5 a	687.5 ± 470.3 a	625.0 ± 357.5 a
3° EPG	1175.0 ± 966.2 a	912.5 ± 394.3 a	700.0 ± 492.8 a	637.5 ± 417.2 a
4° EPG	1187.5 ± 1088.1 a	1062.5 ± 704.9 a	862.5 ± 768.9 a	900.0 ± 755.9 a
5° EPG	1637.5 ± 1391.7 a	1062.5 ± 817.5 a	1050.0 ± 358.5 a	987.5 ± 593.8 a
6° EPG	2087.5 ± 1119.2 a	1875.0 ± 1449.8 a	1700.0 ± 828.0 a	1250.0 ± 307.0 a
7° EPG	1737.5 ± 878.2 a	1137.5 ± 726.9 ab	1562.5 ± 468.8 ab	987.5 ± 432.3 b
8° EPG	1837.5 ± 903.8 a	1625.0 ± 775.9 ab	1537.5 ± 957.5 ab	987.5 ± 445.4 b
9° EPG	2175.0 ± 1578.1 a	1375.0 ± 562.5 ab	1787.5 ± 874.1 ab	1087.5 ± 664.2 b
10° EPG	2950.0 ± 1822.8 a	1600.0 ± 846.8 b	1712.5 ± 579.2 b	1312.5 ± 707.9 b

Means followed by the same letter in the column do not differ significantly (*p* ≥ 0.05, Student’s *t*-test).

**Table 6:** Efficacy (%) of *Metarhizium robertsii* (LCM S15) in aqueous (FUN-A) and oil (FUN-O) formulations under semi-natural conditions, compared to aqueous (CTR-A) and oil (CTR-O) control groups.

	Efficacy (%)	
	FUN-A	FUN-O
1° EPG	0%	0%
2° EPG	41.89%	0%
3° EPG	22.34%	0%
4° EPG	10.52%	0%
5° EPG	35.11%	10.31%
6° EPG	10.17%	26.47%
7° EPG	34.53%	36.8%
8° EPG	13.07%	35.77%
9° EPG	36.78%	39.16%
10° EPG	45.76%	23.35%
Mean efficacy (%)	24.79%	16.57%

**Table 7:** Mean ± standard deviation of larvae recovered using the Baermann technique.

		Number of Larvae	
Groups	1° Baermann	2° Baermann	3° Baermann
FUN-A	91.2 ± 51.0 a	58.9 ± 30.7 a	114.4 ± 24.5 ab
CTR-A	98.4 ± 37.3 a	69.9 ± 24.6 a	147.9 ± 35 a
FUN-O	65.8 ± 18.1 a	44.2 ± 13.2 a	87.1 ± 28.2 b
CTR-O	67.9 ± 47.1 a	62.0 ± 24.8 a	127.3 ± 49.2 ab

Means followed by the same letter in the column do not differ significantly (*p* ≥ 0.05, Student’s *t*-test).

**Table 8:** Efficacy (%) based on the Baermann technique for the following treatments: aqueous control (CTR-A), oil control (CTR-O), aqueous suspension of *Metarhizium robertsii* (LCM S15) (FUN-A), and oil formulation of *M. robertsii* (LCM S15) (FUN-O).

		Efficacy (%)		
Groups	1° Baermann	2° Baermann	3° Baermann	Mean Efficacy
FUN-A	7.33%	15.73%	22.65%	15.23%
FUN-O	3.09%	28.70%	31.57%	21.12%

## Data Availability

The raw data supporting the conclusions of this article will be made available by the authors on request.

## Abbreviations

CONCEA	National Council for the Control of Animal Experimentation
CEUA	Ethics Committee on Animal Use
UFRB	Federal University of Recôncavo da Bahia
LPDP	Laboratory of Parasitology and Parasitic Diseases
HUMV	University Veterinary Medicine Hospital
HMDS	Hexamethyldisilazane

## References

[1] Kour G., Silva T.A.C.C., Walkden-Brown S.W., Baleiverata A., Mala S., Rao R., Prasad D., Cowley F.C. (2025). Assessing anthelmintic resistance on small ruminant farms in a tropical production system. Small Rumin. Res..

[2] IBGE (2022). Censo Agropecuário.

[3] Guimarães M.J.M., Lopes I. Análise da precipitação do município de Cruz das Almas através da técnica de quantis. Proceedings of the XXV Congresso Nacional de Irrigação e Drenagem.

[4] Hassum I.C. (2014). Famacha method as a tool for selective control of nematode parasites in sheep. Rev. Bras. Med. Vet..

[5] Brito D.R.B., Santos A.C.G., Teixeira W.C., Guerra R.M.S.N.C. (2009). Parasitos gastrintestinais em caprinos e ovinos da microrregião do Alto Mearim e Grajaú, no estado do Maranhão, Brasil. Ciênc. Anim. Bras..

[6] Mavundela S., Dzemo W.D., Thekisoe O. (2025). Anthelmintic resistance in gastrointestinal nematodes on communally reared sheep farms of the King Sabata Dalindyebo Municipality, South Africa. Parasitol. Res..

[7] Carson A., Reichel R., Bell S., Collins R., Smith J., Bartley D. (2023). *Haemonchus contortus*: Uma visão geral. Vet. Rec..

[8] Almeida L.R., Castro A.A., Silva F.J.M., Fonseca A.H. (2005). Desenvolvimento, sobrevivência e distribuição de larvas infectantes de nematoides gastrintestinais de ruminantes na estação seca da Baixada Fluminense, RJ. Rev. Bras. Parasitol. Vet..

[9] Wang T., Avramenko R.W., Redman E.M., Wit J., Gilleard J.S., Colwell D.D. (2020). High levels of third-stage larvae (L3) overwinter survival for multiple cattle gastrointestinal nematode species on western Canadian pastures as revealed by ITS2 rDNA metabarcoding. Parasites Vectors.

[10] Silva H.M. (2008). Parasitismo Gastrintestinal em Diferentes Intensidades de Pastejo no Capim Tanzânia, em Caprinos.

[11] Craig T.M. (2018). Gastrointestinal nematodes, diagnosis and control. Vet. Clin. N. Am. Food Anim. Pract..

[12] Braga R.M. (1980). Desenvolvimento e Sobrevivência de ovos de Nematoides de Bovinos sob Condições Naturais.

[13] Lyaku J.R.S., Monrad J., Kassuku A.A. (1988). Larval ecology of bovine strongylid worms in tropical soils: In vitro longevity of infective strongylid larvae in different soil types. Trop. Anim. Health Prod..

[14] Salgado J.A., Santos C.P. (2016). Overview of anthelmintic resistance of gastrointestinal nematodes of small ruminants in Brazil. Rev. Bras. Parasitol. Vet..

[15] Verma R., Lata K., Das G. (2018). An overview of anthelmintic resistance in gastrointestinal nematodes of livestock and its management: India perspectives. Int. J. Chem. Stud..

[16] Manzanilla F.A.H., Robertosa N.F.O., Garduñoc R.G., Sarmientob R.C., Acostab J.F.J.T. (2017). Gastrointestinal nematode populations with multiple anthelmintic resistance in sheep farms from the hot humid tropics of Mexico. Vet. Parasitol. Reg. Stud. Rep..

[17] Borges C.C.L. (2003). Atividade in vitro de anti-helmínticos sobre larvas infectantes de nematódeos gastrintestinais de caprinos, utilizando a técnica de coprocultura quantitativa (Ueno, 1995). Parasitol. Latinoam..

[18] Amorim V.R. (2020). Uso do Tratamento Seletivo Como Método de Controle das Parasitoses Gastrintestinais de Caprinos no Brejo Paraibano.

[19] Illanes F.A., Romero J.R., Lauroua C., Pruzzo C.I., DI Paolo L.A., Escapil J.M. (2021). Resistencia antihelmíntica en un tambo caprino en la Provincia de Buenos Aires. Rev. Med. Vet..

[20] Fernandes F.O., Sousa S.T.P., Cavalcante I.C.A., Santos L.C., Coelho M.S., Donato L.E. (2021). Avaliação do uso de anti-helmíntico convencional de forma isolada comparada ao uso associado com fitoterápico *Allium sativum* L. no tratamento de verminoses em caprinos: Resumo científico (Mensão honrosa). VET—Resumos Apresentados em Eventos.

[21] Batista I.L., Costa J.N.P., Sousa R.A., Mendonça I.L. (2022). Resistência anti-helmíntica aos benzimidazóis em parasitos de animais de produção. Ciênc. Anim..

[22] Soares S.C.P. (2023). Resistência de nematoides gastrintestinais de caprinos e ovinos aos anti-helmínticos levamisol, ivermectina e albendazol. Ciênc. Anim. Bras..

[23] Beys-da-Silva W.O., Rosa R.L., Berger M., Coutinho-Rodrigues C.J.B., Vainstein M.H., Schrank A., Bittencourt V.R.E.P., Santi L. (2020). Updating the application of *Metarhizium anisopliae* to control cattle tick *Rhipicephalus microplus* (Acari: Ixodidae). Exp. Parasitol..

[24] Garcia M.V., Andreotti R., Koller W.W., Andreotti R., Garcia M.V., Koller W.W. (2019). Biologia e importância do carrapato *Rhipicephalus (Boophilus) microplus*. Carrapatos na Cadeia Produtiva de Bovinos.

[25] Mota M.A., Campos A.K., Araújo J.V. (2003). Controle biológico de helmintos parasitos de animais: Estágio atual e perspectivas futuras. Pesq. Vet. Bras..

[26] Fausto G.C., Fausto M.C., Vieira Í.S., Freitas S.G., Carvalho L.M., Oliveira I.C., Silva E.N., Campos A.K., Araújo J.V. (2021). Formulation of the nematophagous fungus *Duddingtonia flagrans* in the control of equine gastrointestinal parasitic nematodes. Vet. Parasitol..

[27] Rodrigues J.A., Roque F.L., Álvares F.B.V., Silva A.L.P.D., Lima E.F., Silva Filho G.M.D., Feitosa T.F., Araújo J.V., Braga F.R., Vilela V.L.R. (2021). Efficacy of a commercial fungal formulation containing *Duddingtonia flagrans* (Bioverm^®^) for controlling bovine gastrointestinal nematodes. Rev. Bras. Parasitol. Vet..

[28] Rodrigues J.A., Roque F.L., Lima B.A., Silva Filho G.M., Oliveira C.S.M., Sousa L.C., Silva A.L.P., Lima E.F., Feitosa T.F., Braga F.R. (2022). Control of sheep gastrointestinal nematodes on pasture in the tropical semiarid region of Brazil using Bioverm^®^ (*Duddingtonia flagrans*). Trop. Anim. Health Prod..

[29] Mendes L.Q., Ferraz C.M., Ribeiro N.R.C., Ulfeldt K.B., Ribeiro J.C.C., Merizio M.F., Rossi G.A.M., Aguiar A.A.R.M., Araújo J.V., Soares F.E.F. (2023). Efficacy of *Duddingtonia flagrans* (Bioverm^®^) on the biological control of buffalo gastrointestinal nematodes. Exp. Parasitol..

[30] Nunes G.T., Corrêa D.C., Chitolina M.B., Da Rosa G., Pereira R.C.D.F., Cargnelutti J.F., Vogel F.S.F. (2023). Efficacy evaluation of a commercial formulation with *Duddingtonia flagrans* in equine gastrointestinal nematodes. J. Equine Vet. Sci..

[31] Silva M.E., Uriostegui M.A.M., Millán-Orozco J., Gives P.M., Hernández E.L., Braga F.R., Araújo J.V. (2017). Predatory activity of *Butlerius* nematodes and nematophagous fungi against *Haemonchus contortus* infective larvae. Rev. Bras. Parasitol. Vet..

[32] Alves S.B., Alves S.B. (1998). Fungos entomopatogênicos. Controle Microbiano de Insetos.

[33] Murad A.M., Laumann R.A., Lima T.A., Sarmento R.B.C., Noronha E.F., Rocha T.L., Franco O.L. (2006). Screening of entomopathogenic *Metarhizium anisopliae* isolates and proteomic analysis of secretion synthesized in response to cowpea weevil (*Callosobruchus maculatus*) exoskeleton. Comp. Biochem. Physiol. C Toxicol. Pharmacol..

[34] Santi L., Silva W.O.B., Pinto A.F.M., Schrank A., Vainstein M.H. (2010). *Metarhizium anisopliae* host–pathogen interaction: Differential immunoproteomics reveals proteins involved in the infection process of arthropods. Fungal Biol..

[35] Barbieri A., Rico I.B., Silveira C., Feltrin C., Dall Agnol B., Schrank A., Lozina L., Klafke G.M., Reck J. (2023). Field efficacy of *Metarhizium anisopliae* oil formulations against *Rhipicephalus microplus* ticks using a cattle spray race. Ticks Tick. Borne Dis..

[36] Velázquez-Sarmiento F., Rodríguez-Vivas R.I., Alonso-Díaz M.A., Fernández-Salas A., Romero-Salas D. (2024). *Metarhizium anisopliae* sensu lato native to livestock soils causes high mortality on *Rhipicephalus microplus* larvae, adults and affects their reproduction. J. Parasitol..

[37] Perinotto W.M.S., Angelo I.C., Golo P.S., Camargo M.G., Quinelato S., Sá F.A., Coutinho-Rodrigues C.J.B., Marciano A.F., Monteiro C.M.O., Bittencourt V.R.E.P. (2017). In vitro pathogenicity of different *Metarhizium anisopliae* s.l. isolates in oil formulations against *Rhipicephalus microplus*. Biocontrol Sci. Technol..

[38] Mascarin G.M., Lopes R.B., Delalibera Í., Fernandes É.K.K., Luz C., Faria M. (2019). Current status and perspectives of fungal entomopathogens used for microbial control of arthropod pests in Brazil. J. Invertebr. Pathol..

[39] Rodrigues M.L.A. (2016). Classificação e Morfologia de Nematóides em Medicina Veterinária.

[40] Moura I.A., Pereira I.S., Pires R., Ribeiro O.L., Monteiro C.M.O., Rocha L.S., Perinotto W.M.S. (2023). First report of *Metarhizium anisopliae* s.l. action on gastrointestinal ruminant nematodes in free-living stage and its persistence in soil. Biocontrol Sci. Technol..

[41] Rodrigues M.L.A., Bittencourt V.R.E.P., Anjos D.H.S., Castro A.A. (1996). Efeito do fungo *Metarhizium anisopliae* sobre os estádios pré-parasíticos de *Cyathostominae* (Nematoda: Strongylidae). Ciênc. Rural.

[42] Fernandes E.K.K., Keyser C.A., Rangel D.E.N., Foster R.N., Roberts D.W. (2010). CTC medium: A novel dodine-free selective medium for isolating entomopathogenic fungi, especially *Metarhizium acridum*, from soil. Biol. Control.

[43] Bischoff J.F., Rehner S.A., Humber R.A. (2009). A multilocus phylogeny of the *Metarhizium anisopliae* lineage. Mycologia.

[44] Driver F., Milner R.J., Trueman J.W.H. (2000). A taxonomic revision of *Metarhizium* based on a phylogenetic analysis of rDNA sequence data. Mycol. Res..

[45] Tulloch M. (1976). The genus *Metarhizium*. Trans. Br. Mycol. Soc..

[46] Gordon H.M., Whitlock H.N. (1939). A new technique for counting nematode eggs in sheep faeces. J. Counc. Sci. Ind. Res. Aust..

[47] Keith R.K. (1953). The differentiation of the infective larvae of some common nematode parasites of cattle. Aust. J. Zool..

[48] Aw K.M., Hue S.M. (2017). Mode of Infection of *Metarhizium* spp. Fungus and Their Potential as Biological Control Agents. J. Fungi.

[49] Arruda W., Lübeck I., Schrank A., Vainstein M.H. (2005). Morphological alterations of *Metarhizium anisopliae* during penetration of *Boophilus microplus* ticks. Exp. Appl. Acarol..

[50] Faria L.E.M., Ferreira O.B.A.S., Machado A.L., Costa J.N., Perinotto W.M.S. (2022). Monitoring environmental conditions on the speed of development and larval migration of gastrointestinal nematodes in *Urochloa decumbens* in northeastern Brazil. Vet. Parasitol. Reg. Stud. Rep..

[51] Silva H.M. (2014). Nematodioses gastrintestinais de caprinos: Uma revisão. Rev. Ciênc. Agroveterin..

[52] Amarante A.F.T. (2014). Os Parasitas de Ovinos.

[53] Paixão F.R.S., Muniz E.R., Barreto L.P., Bernardo C.C., Mascarin G.M., Luz C., Fernandes K.K. (2017). Tolerância ao calor aumentada proporcionada por formulações de conídios à base de óleo de *Metarhizium anisopliae* e *Metarhizium robertsii*. Biocontrol Sci. Technol..

[54] Alves F.M., Bernardo C.C., Paixão F.R.S., Barreto L.P., Luz C., Humber R.A., Fernandes É.K.K. (2017). Heat-stressed *Metarhizium anisopliae*: Viability (in vitro) and virulence (in vivo) assessments against the tick *Rhipicephalus sanguineus*. Parasitol. Res..

[55] Prior C., Jollands P., Le Patourel G. (1988). Infectivity of oil and water formulations of *Beauveria bassiana* (Deuteromycotina: Hyphomycetes) to the cocoa weevil pest *Pantorhytes plutus* (Coleoptera: Curculionidae). J. Invertebr. Pathol..

[56] Camargo M.G., Marciano A.F., Sá F.A., Perinotto W.M.S., Quinelato S., Golo P.S., Angelo I.C., Prata M.C.A., Bittencourt V.R.E.P. (2014). Commercial formulation of *Metarhizium anisopliae* for the control of Rhipicephalus microplus in a pen study. Vet. Parasitol..

[57] Camargo M.G., Golo P.S., Angelo I.C., Perinotto W.M.S., Sá F.A., Quinelato S., Bittencourt V.R.E.P. (2012). Effect of oil-based formulations of acaripathogenic fungi to control *Rhipicephalus microplus* ticks under laboratory conditions. Vet. Parasitol..

[58] Camargo M.G., Nogueira M.R.S., Marciano A.F., Perinotto W.M.S., Coutinho-Rodrigues C.J.B., Scott F.B., Angelo I.C., Prata M.C.A., Bittencourt V.R.E.P. (2016). *Metarhizium anisopliae* for controlling *Rhipicephalus microplus* ticks under field conditions. Vet. Parasitol..

[59] Perinotto W.M.S., Camargo M.G., Golo P.S., Angelo I.C., Quinelato S., Monteiro C.M.O., Sá F.A., Coutinho-Rodrigues C.J.B., Marciano A.F., De Paulo J.F. (2013). Controle de *Dermacentor nitens* utilizando uma formulação comercial à base de *Metarhizium anisopliae*. Rev. Bras. Med. Vet..

[60] Vincent C., Hallman G., Panneton B., Fleurat-Lessard F. (2003). Management of agricultural insects with physical control methods. Annu. Rev. Entomol..

[61] Walstad J.D., Anderson R.F., Stambaugh W.J. (1970). Effects of environmental conditions on two species of muscardine fungi (*Beauveria bassiana* and *Metarhizium anisopliae*). J. Invertebr. Pathol..

[62] Barreto L.P., Luz C., Mascarin G.M., Roberts D.W., Arruda W., Fernandes É.K.K. (2016). Effect of heat stress and oil formulation on conidial germination of *Metarhizium anisopliae* s.s. on tick cuticle and artificial medium. J. Invertebr. Pathol..

[63] Alves S.B., Nogueira N.L. (1984). Efeito da temperatura na germinação e viabilidade de *Metarhizium anisopliae* (Metsch.) Sorokin. Anais do Congresso Brasileiro de Entomologia.

[64] Mesquita E., Hu S., Lima T.B., Golo P.S., Bidochka M.J. (2023). Utilization of *Metarhizium* as an insect biocontrol agent and a plant bioinoculant with special reference to Brazil. Front. Fungal Biol..

[65] Sullivan C.F., Parker B.L., Skinner M. (2022). A review of commercial *Metarhizium*- and *Beauveria*-based biopesticides for the biological control of ticks in the USA. Insects.

